# A Comparison of Co-expression Networks in Silk Gland Reveals the Causes of Silk Yield Increase During Silkworm Domestication

**DOI:** 10.3389/fgene.2020.00225

**Published:** 2020-03-27

**Authors:** Qiu-Zhong Zhou, Ping Fu, Shu-Shang Li, Chang-Jiang Zhang, Quan-You Yu, Chuan-Zhen Qiu, Hong-Bo Zhang, Ze Zhang

**Affiliations:** ^1^Laboratory of Evolutionary and Functional Genomics, School of Life Sciences, Chongqing University, Chongqing, China; ^2^Postdoctoral Station of Biomedical Engineering, Chongqing University, Chongqing, China

**Keywords:** silkworm, domestication, silk gland, silk yield, transcriptome, co-expression

## Abstract

Long-term domestication and selective breeding have increased the silk yield of the domestic silkworm (*Bombyx mori*) by several times the amount of the silk yield of its wild ancestor (*Bombyx mandarina*). However, little is known about the molecular mechanisms behind the increase in silk yield during domestication. Based on dynamic patterns of functional divergence in the silk gland between domestic and wild silkworms, we found that at early and intermediate stages of silk gland development, the up-regulated genes of the domestic silkworm were mainly involved in DNA integration, nucleic acid binding, and transporter activity, which are related to the division and growth of cells. This has led to the posterior silk gland (PSG) of the domestic silkworm having significantly more cells (“factories” of fibroin protein synthesis) than that of the wild silkworm. At the late stage of silk gland development, the up-regulated genes in the domestic silkworm was enriched in protein processing and ribosome pathways, suggesting protein synthesis efficiency is greatly improved during silkworm domestication. While there was an increase in fibroin protein synthesis, the production of sericin protein was simultaneously reduced in the silk gland of the domestic silkworm. This reflects that domestic and wild silkworms have been under different selection pressures. Importantly, we found that the network co-expressed with the silk-coding genes of the domestic silkworm was larger than that of the wild silkworm. Furthermore, many more genes co-expressed with silk-coding genes in the domestic silkworm were subjected to artificial selection than those in the wild silkworm. Our results revealed that the increase of silk yield during silkworm domestication is involved in improvement of a biological system which includes not only expansion of “factories” (cells of PSG) of protein synthesis, but also a high expression of silk-coding genes and silk production-related genes such as biological energy, transport, and ribosome pathway genes.

## Introduction

The domestic silkworm (*Bombyx mori*) was domesticated from the Chinese wild silkworm (*Bombyx mandarina*) about 5,000 years ago (Astaurov and Rovinskaya, 1959; Shimada et al., 1995; Sun et al., 2012). As an economical insect, silk produced by the domestic silkworm is an important material not only for textiles and industrial application, but also for biomaterials and cosmetics (Goldsmith et al., 2005). Sericulture remains a major source of income for farmers in some developing countries, especially China and India. Silk, composed of fibroin and sericin proteins, is synthesized in the silk gland, a silk-producing organ of the silkworm (Xia et al., 2014). Because of its economic importance, the composition and genetic basis of silk in the domestic silkworm have been extensively studied up to now.

Silk fibroin consists of the fibroin heavy chain (*Fib-H*), fibroin light chain (*Fib-L*), and 25-kD polypeptide proteins (*P25*) with a molar ratio of 6:6:1 (Inoue et al., 2000), which are synthesized in the posterior silk gland (PSG), one of three specialized compartments of the gland. Sericin synthesized in the middle silk gland (MSG) is mainly composed of a variety of glue proteins including *Sericin1*, *Sericin2*, and *Sericin3*. During the past few decades, the genes encoding silk fibroin (*Fib-H*, *Fib-L*, and *P25*) and sericin (*Sericin1*, *Sericin2*, and *Sericin3*) have been identified and cloned (Gamo, 1982; Chevillard et al., 1986; Michaille et al., 1986; Yamaguchi et al., 1989; Zhou et al., 2000). Although the genetic loci underlying the variation of silk yield among different domestic silkworm strains have been mapped, the candidate genes responsible for silk yield remain to be identified (Zhan et al., 2009; Li et al., 2013, 2015; Li C. et al., 2017). Later, the comparative analysis of transcriptomes among the domestic silkworm strains with different silk yields was used to identify the genes associated with silk yield (Li et al., 2016; Luan et al., 2018). It was found that the differentially expressed genes (DEGs) were mainly involved in the processing and biosynthesis of proteins (Li et al., 2016), and silk gland development or protein synthesis (Luan et al., 2018). Although these studies have provided some insights into the genetic basis of silk production, no gene that regulates silk yield in silkworm has been functionally verified. This implies that the molecular mechanisms underlying silk production may be much more complex than thought before.

Compared with its wild ancestor, long-term artificial breeding and selection have led to the domestic silkworm having very different phenotypes. Silk yield of the domestic silkworm is 3–5 times higher than that of the wild silkworm (Fang et al., 2015), Thus, illuminating the molecular mechanisms of the difference in silk yield between domestic and wild silkworms is important not only for improvement of complex traits but also for evolutionary biology. With a transcriptome comparison of silk glands at day 3 of fifth instar larva between domestic and wild silkworms, we identified sixteen up-regulated genes in the domestic silkworm which were related to secretion of proteins, tissue development, and metabolism (Fang et al., 2015). In addition, a shotgun proteomics approach with label-free quantification analysis was used to compare proteomics of PSGs between the domestic and wild silkworms, in which 50 differentially expressed proteins were identified (Li J. Y. et al., 2017). Our recent study also demonstrated that there is a big difference in the abundance of sericin proteins in cocoon between domestic and wild silkworms (Dai et al., 2019), However, these comparisons of the transcriptomes or proteomes were only based on one stage or two stages of silk gland development or cocoon (Fang et al., 2015; Li J. Y. et al., 2017; Dai et al., 2019). The dynamic divergence of silk protein synthesis and regulation in the silk gland between domestic and wild silkworms remains to be investigated.

In this study, we first obtain time-series transcriptome data for silk gland development across domestic and wild silkworms. Then, we investigate dynamic patterns of functional divergence in the silk gland between domestic and wild silkworms. Our results provide some new insights into the causes of silk yield increase during silkworm domestication.

## Materials and Methods

### Phenotypic Investigation of Silk in the Domestic and Wild Silkworms

Wild silkworms were collected in Chongqing, China. The domestic silkworms (Xiafang strain) were obtained from the sericultural research institute of Nanchong. The wild silkworms were reared as previously described (Fang et al., 2015), at 25 ± 1°C and 75 ± 3% relative humidity. Firstly, we surveyed and compared the weight of cocoon silk between the domestic and wild silkworms—each species includes 16 cocoons. Secondly, the cell number of the PSG was determined in the domestic and wild silkworms by nucleus staining with 4′,6-diamidino-2-phenylindole (DAPI, a fluorescent stain) (Zhang et al., 2012). Moreover, we used the scanning electron microscopy to compare the microstructural morphology of silk fibers in the cross-section between the domestic and wild silkworms (Chen et al., 2012; Guan et al., 2017). The cocoon was divided into three layers: inner, middle, and outer (Zhang et al., 2013). The bundled silk fibers of each layer were put through a small tube and cut into the short section, and then sputter-coated with palladium for 2 min (Guan et al., 2017). The small tubes fixing onto the platform were used for the cross-sectional view of the silk fibers with the SU3500 (Hitachi, Japan).

### Sample Preparation, RNA Extraction, and RNA Sequencing

**FIGURE 1:**
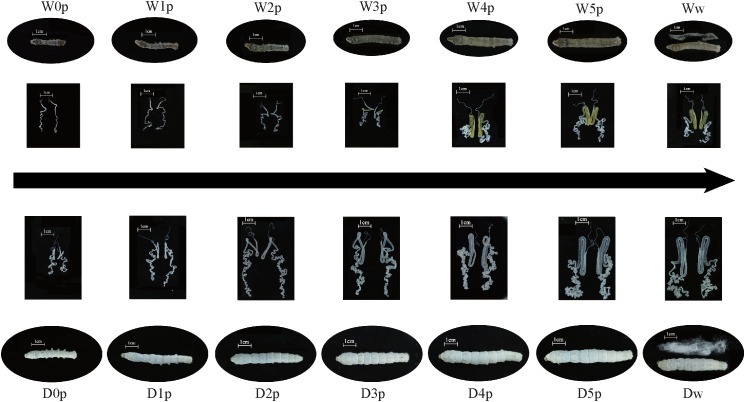
Morphology variation of the fifth instar larva and silk gland between the domestic and wild silkworms. W, wild silkworm; D, domestic silkworm (Xiafang strain); p, time point; w, the wandering stage of silkworm (the last time point of the fifth instar); 0p, start of the fifth instar.

The fifth instar duration is longer in the domestic silkworm than the wild silkworm. Thus, the silk gland of the domestic silkworm was dissected at the fourth molt (D0p), the 28 h of the fifth instar (D1p), the 56 h of the fifth instar (D2p), the 84 h of the fifth instar (D3p), the 112 h of the fifth instar (D4p), the 140 h of the fifth instar (D5p), and the wandering stage (Dw). The silk gland of the wild silkworm was dissected at the fourth molt (W0p), the 24 h of the fifth instar (W1p), the 48 h of the fifth instar (W2p), the 72 h of the fifth instar (W3p), the 96 h of the fifth instar (W4p), the 120 h of the fifth instar (W5p), and the wandering stage (Ww) (Figure 1). All the silk gland samples were immediately frozen in liquid nitrogen for further investigation. Total RNA was extracted using TRIzol reagent (Invitrogen, Carlsbad, CA, United States) following the manufacturer’s instructions. 1.5 μg of total RNA was used to produce barcoded RNA sequencing libraries using the NEBNext^®^ UltraTM RNA Library Prep Kit (NEB, United States). Libraries were pooled in five different pools based on barcode compatibility, and in each pool 150 bp pair-end RNA sequencing was performed on the Illumina platform.

### Quality Control and Transcriptome Assembly

The reads containing the adapter, or N bases >10%, or low-quality base (quality scores ≤5) >50% were filtered from the raw data by the Perl program. The first 25 bp of the reads was trimmed because of the variation of per base sequence content and mapped to the reference genome of the silkworm from the Ensembl database release 31 (*B. mori*: GCA_000151625) with TopHat2 v2.1.1, respectively (Kim et al., 2013). The transcripts of silk gland were assembled in each sample using Cufflinks v2.2.1 with the option -N 3 −read-gap-length 3 −read-edit-dist 3 –G (Kim et al., 2013; Patro et al., 2014).

### Quantification of Gene Expression and Identification of Differentially Expressed Genes

FPKM (Fragments Per Kilobase of transcript per Million mapped reads) is used to estimate the level of gene expression. To ensure the precision of expression, only the uniquely mapped reads were allowed to quantify the gene expression. The read counts of gene were calculated in the 25 developmental samples using the software HTseq v0.6.0 (Anders et al., 2014). The read counts were standardized by the R package (edgeR) with the method of TMM (the weighted trimmed mean of *M*-values) and then transformed to the FPKM based on the gene length and sequencing depth (Robinson and Oshlack, 2010; Robinson et al., 2010). For the biological replicates, the mean of FPKM is chosen as the transcriptional signal for each gene. RNA-seq of tissues was performed with the same pipeline (Wu et al., 2016).

The differential expression analysis was conducted between the domestic and wild silkworms using the edgeR at the stage of 0p, 1p, 2p, 3p, 4p, 5p, and w, respectively (Robinson et al., 2010). The *P*-value was adjusted by the Benjamini–Hochberg (BH) method (Benjamini and Hochberg, 1995). The adjusted *P*-value < 0.05 and | log2 fold-change| >1 are defined as significantly differential expression for the replicated sample (D0p vs. W0p, D1p vs. W1p, D2p vs. W2p, D3p vs. W3p, and D5p vs. W5p). The criteria of adjusted *P*-value < 0.01 and | log2 fold-change| >1 are for the non-replicated sample (D4p vs. W4p and Dw vs. Ww).

### Comparative Analysis of Co-expression Network

The weighted gene co-expression network analysis was carried out with the R package WGCNA v1.51 (Langfelder and Horvath, 2008). Since the lowly expressed or non-varying genes usually represent the “noise” for the co-expression network analysis, the genes with FPKM < 1 in more than 10 samples were filtered out (Langfelder and Horvath, 2008; Miller et al., 2010; Robinson et al., 2010). And the non-varying genes were also removed using the “goodSamplesGenes” function of WGCNA with the default parameter (Langfelder and Horvath, 2008; Miller et al., 2010). Gene expression data were used to generate the similarity matrix of Pearson correlations between gene and gene (*s*_*ij*_ = |*cor*(*x*_*i*_*x*_*j*_)|) across all examined samples in the domestic and wild silkworms, respectively (Langfelder and Horvath, 2008). The adjacency matrices were created using the adjacency function with a weighted soft threshold ($a_{i\,j}=s_{i\,j}^{\beta },\beta =16$). The weighted soft threshold (β) was estimated by the criterion of approximate scale-free topology (Zhang and Horvath, 2005). To weaken the effect of “noise” connections, the adjacency matrices were transformed into the topological overlap matrixes (TOM) using the “TOMsimilarity” function of WGCNA (Langfelder and Horvath, 2008). 1-TOM was used to perform average linkage hierarchical clustering and for module detection (Ravasz et al., 2002; Yang et al., 2018). Fold change of weight value >1.5 was defined as differential co-expression between the domestic and wild silkworms (Lu et al., 2016).

## Functional Annotation and Enrichment Analysis

A TransDecoder was used to predict the gene protein and only the longest one was retained (Grabherr et al., 2011). All the genes were annotated for protein function performing by the InterProScan (v60) (Finn et al., 2017). The results of InterProScan were transformed into gene ontology (GO) annotations. We carried out the GO enrichment for DEGs and module genes with GOseq (Young et al., 2010). KEGG (Kyoto Encyclopedia of Genes and Genomes) pathway analysis was performed using KOBAS 3.0 based on the hypergeometric test and Benjamini–Hochberg correction (Xie et al., 2011).

### Population Genetic Analysis

We performed the whole-genome resequencing for 10 domestic silkworms and 8 wild silkworms. The domestic silkworms were the 7532, Jianpuzhai, HB05, S02, S03, N4, Xiafang, Xianghui, Xiaoshiwan, and Yanjinhuang strains and the wild silkworms were obtained from Ankang⋅Shanxi, Anyue⋅Sichuan, Beibei⋅Chongqing, Hongya⋅Sichuan, Nanchong⋅Sichuan, Suzhou⋅Jiangsu, Ziyang⋅Sichuan, and Wuhan⋅Hubei. Postnatally, we mapped reads of per sample to the reference genome using the software BWA-MEM (v0.7.7) (Li and Durbin, 2010). The processes, including sorting, duplicate marking, local realignment, and base quality recalibration were then conducted with the alignment file (bam format) (McKenna et al., 2010). The calibrated alignment file was used to identify the SNP. The SNP was discovered using the tools including GATK, SAMtools v1.4, and freebayes v1.0.2, respectively (Li et al., 2009; McKenna et al., 2010; Garrison and Marth, 2012). The SNP, which is simultaneously identified by GATK, SAMtools, and freebayes, is defined as a high-quality SNP. Only the high-quality SNP was selected for the next process. Nucleotide polymorphism (π) and fixation index (*Fst*) of population differentiation were calculated using a sliding window analysis (Danecek et al., 2011). The window (π*_*D*_*/π*_*w*_* or π*_*D*_*-π*_*w*_* < 5% value of empirical distribution and *Fst* > 95% value of empirical distribution) is identified as the genomic regions harboring footprints of artificial selection in domestic silkworms (Xia et al., 2009; Qiu et al., 2015; Wang et al., 2016). The gene overlapped with a genomic region of artificial selection is identified as an artificial selection gene (Qiu et al., 2015; Wang et al., 2016).

### Quantitative Real-Time PCR

Sequence-specific primers of fibroin genes were designed using primer-blast and the specificity of the primers is evaluated by the *B. mori* genomic sequence (taxid: 7091) (Supplementary Table S1; Ye et al., 2012). Quantitative real-time PCR (qRT-PCR) was conducted using the CFX96^TM^Real-Time PCR Detection System (Bio-Rad, United States) and SYBR Green qRT-PCR Mix (Bio-Rad, United States). The relative expression levels of fibroin genes were normalized against the corresponding ribosomal protein L3.

## Results

### Assembly of Silk Gland Transcriptomes

For both male and female, the weight of the larva, whole cocoon, pupa, and the cocoon’s shell of the domestic silkworm (Xiafang strain, XF) are significantly higher compared to that of the wild silkworm (Supplementary Figure S1). Especially, the cocoon shell weight (CSW) of the domestic silkworm is about 10-fold of the CSW of the wild silkworm. The silk gland is well known as the most important organ for silk protein synthesis (Goldsmith et al., 2005). To further find if there is any transcriptional divergence during silk gland development between domestic and wild silkworms, we carried out the pair-end RNA-seq at seven developmental stages of silk gland and comprehensively characterized the gene expression dynamics in the silk glands of domestic and wild silkworms. After removing the low-quality reads, 559,843,084 and 545,393,238 clean reads are obtained from the domestic and wild silkworms, respectively (Supplementary Table S2). The clean data are mapped to the silkworm genome and then assembled into the 29,691 gene loci using the tools Tophat2-Cufflinks v.2.2.1 (Trapnell et al., 2012). Then we assessed the gene expression levels based on the uniquely mapped reads of gene loci (Supplementary Table S3). The gene expression patterns are similar between domestic and wild silkworms (Supplementary Figure S2). More than 67.5% of genes show low expression levels (0 ≤ FPKM < 1) in domestic and wild silkworms (Supplementary Table S4). Only 95∼125 and 101∼127 genes present super high expression levels (>1,000 FPKM) in the domestic and wild silkworms, respectively. Specifically, both sericin and fibroin genes are very highly expressed in the domestic and wild silkworms (Supplementary Table S3), indicating that the function of the silk gland is highly conserved between both (Fang et al., 2015).

### Divergence of Transcriptional Level During Silk Gland Development Between the Domestic and Wild Silkworms

**FIGURE 2:**
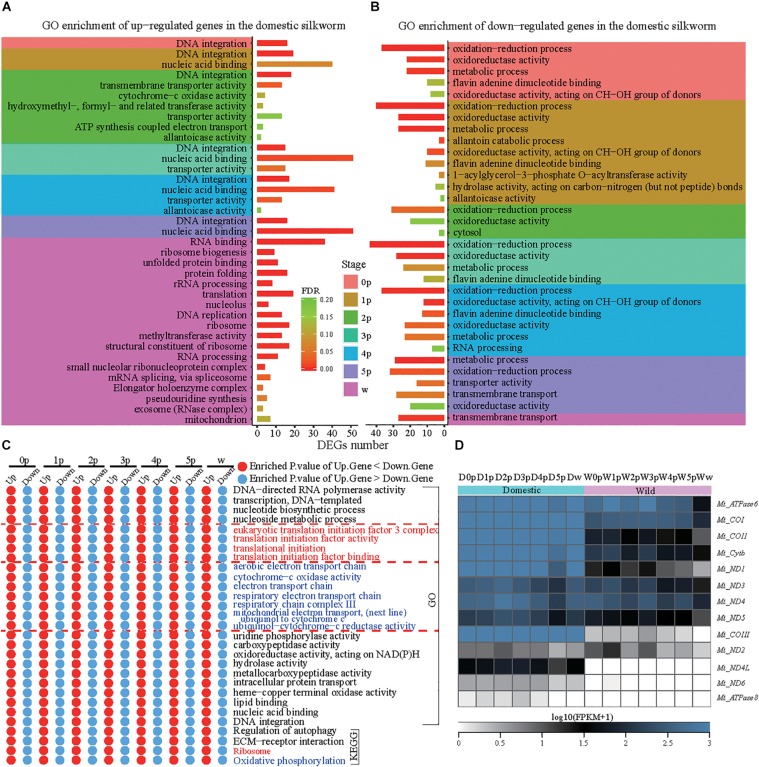
Functional divergences in the silk gland development between the domestic and wild silkworms. **(A)** Gene ontology enrichment of up-regulated genes in the domestic silkworm. **(B)** Gene ontology enrichment of down-regulated genes in the domestic silkworm. **(C)** The GO and KEGG terms exhibit higher enrichment in the up-regulated genes than the down-regulated genes at all time points. **(D)** Expression profile of all mitochondrial genes in the domestic and wild silkworms.

To compare the dynamic process of silk gland development, we identified DEGs at seven developmental stages between the domestic and wild silkworms (Figure 1). Finally, 1,282, 1,149, 1,215, 1,291, 1,076, 1,139, and 1,264 genes are identified as DEGs between the domestic and wild silkworms at the time points of 0p, 1p, 2p, 3p, 4p, 5p, and wandering stage (w), respectively (Supplementary Table S5). The up-regulated genes of the domestic silkworm are prominently enriched in the DNA integration, nucleic acid binding, and transporter activity (Figure 2A). The genes related to transporter activity (such as BGIBMGA004507, BGIBMGA004510, BGIBMGA012890, and XLOC_018800) are up-regulated in the domestic silkworm at least at five time points (Supplementary Figure S3A). Furthermore, the pathway of extracellular matrix (ECM) receptors’ interaction is strongly associated with the up-regulated genes which are mediated by transmembrane molecules such as integrins and proteoglycans (Supplementary Figure S3B). The integrins and proteoglycans such as BGIBMGA000915, BGIBMGA001498, and BGIBMGA002430 play important roles in controlling cellular activities and neurotransmitter release, which present relatively high expression in the domestic silkworm at most of the developmental stages (Supplementary Figure S3B). These results suggest that the exchanges of biomolecules and signals in the cells of the silk gland were more frequent in the domestic silkworm. In the late stage, the up-regulated genes in the domestic silkworm display a strong enrichment in the genes related to protein processing in the endoplasmic reticulum and the ribosome (Figure 2A and Supplementary Figure S4A). The down-regulated genes are involved in oxidative-reduction, oxidoreductase activity and metabolic pathways (Figure 2B and Supplementary Figure S4B), which are related to antioxidant systems. These results are consistent with the previous report (Fang et al., 2015).

To further examine the functional shift between the domestic and wild silkworm, we identified the functional term which is more or less enriched at all time points (Figure 2C and Supplementary Figure S5). The up-regulated genes revealed more enrichment in the translational initiation and ribosome pathway at all examined time points (Figure 2C). Most of the ribosome genes are highly expressed in the domestic silkworm especially at the late stages of silk gland development (Supplementary Figure S6). This indicates that protein synthesis is more active in the domestic silkworm than in the wild silkworm. Increased activity of protein synthesis will consume more biological energy (Ma et al., 2011). Interestingly, the electron transport chain presents more enrichment in the up-regulated genes during silk gland development. The expression of mitochondrial genes such as *Mt_ATPase6, Mt_COI*, and *Mt_COII* is higher in the silk gland of the domestic silkworm (Figure 2D). Since the PSG cell is known as the “factory” of fibroins synthesis (Xia et al., 2014), we stained the cells of the posterior silk gland with DAPI, a fluorescent stain (Supplementary Figures S7A,B), and found that the cell number in the silk gland of the domestic silkworm is significantly higher compared to wild silkworm (491 ± 24 vs. 338 ± 20, *P* = 8.34E-11, *t*-test, Supplementary Figure S7C). This indicates that there are more “factories” in the silkworm’s PSG for the synthesis of silk fibroins after domestication and breeding improvement.

### Expression Patterns of Silk-Coding Genes During Silk Gland Development in the Domestic and Wild Silkworms

**FIGURE 3:**
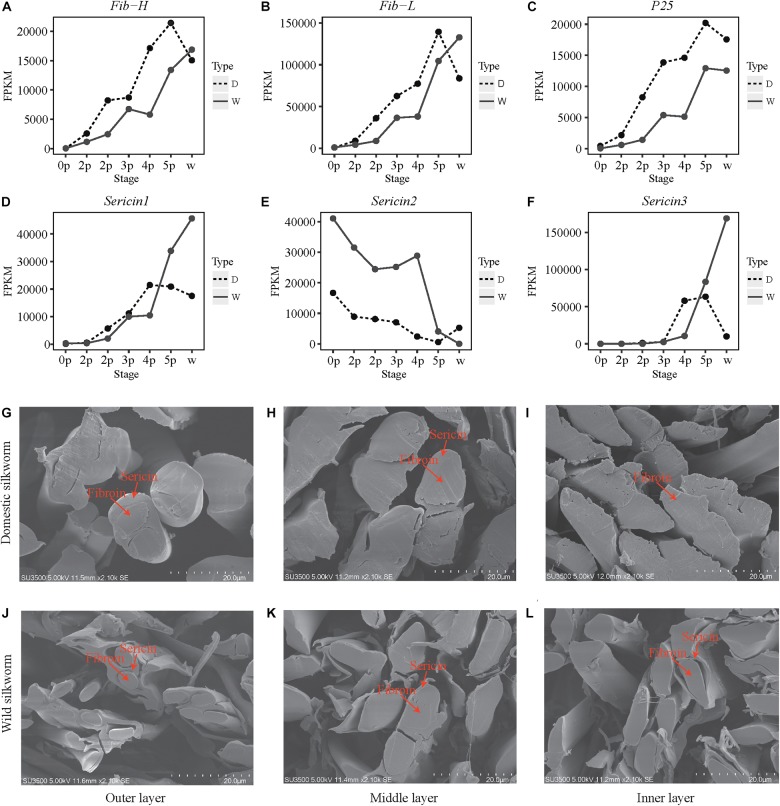
Expression patterns of silk-coding genes and micro-composition of silk between the domestic and wild silkworms. **(A–F)** Expression profile for *Fib-H, Fib-L, P25, Sericin1, Sericin2*, and *Sericin3*. *Y*-axis, the value of FPKM. *X*-axis, the different stages. **(G–I)** Cross-section of the outer, middle, and inner layer silk of the domestic silkworm, respectively. **(J–L)** Cross-section of the outer, middle, and inner layer silk of the wild silkworm, respectively.

Given that an increase in protein synthesis is driven by the increase in transcripts of silk-coding genes, we compared the gene expression levels at development stages and we found that fibroin gene expression is higher in the domestic silkworm at all the seven stages. The result was further confirmed by the RT-qPCR (Figures 3A–C and Supplementary Figure S8). This indicates that the transcription of fibroin genes in the silk gland is enhanced to provide a sufficient template for high-efficient protein synthesis in the domestic silkworm. However, the expression patterns of sericin genes are different from the fibroin genes (Figures 3D–F). *Sericin1* and *Sericin3* genes present continuously increased expression patterns with silk gland development and reached to the highest level at the late stage in the wild silkworm. In contrast, with silk gland development, the expression level of *Sericin2* decreases, and exhibits lower expression level in the domestic than the wild silkworm. These results imply that the silk gland of the domestic silkworm produces more fibroins but less sericins compared to the wild silkworm. To further confirm these results, we inspected the microstructural morphology of a silk fiber cross-section with scanning electron microscopy. The microstructural morphology of the silk fibers of the domestic silkworm exhibit larger fibroins than the wild silkworm’s in the outer, middle, and inner layers of cocoon silk (Figures 3G,H,J,K). At the late stage, the inner layer of cocoon silk fiber produced more sericins by which the fibroins are surrounded in the wild silkworm (Figures 3I,L). This result is consistent with the expression patterns of *sericins* during silk gland development. Moreover, our previous study also found that the cocoon of the domestic silkworm contained more silk fibroins but less sericins than wild the silkworm cocoon, based on the comparative proteomics approach (Dai et al., 2019). These results highlight that the synthetic capacity of fibroin proteins increases while the synthetic capacity of sericin proteins decreases during silkworm domestication.

### Co-expression Network Analysis of the Silk-Coding Genes in Domestic and Wild Silkworms

**FIGURE 4:**
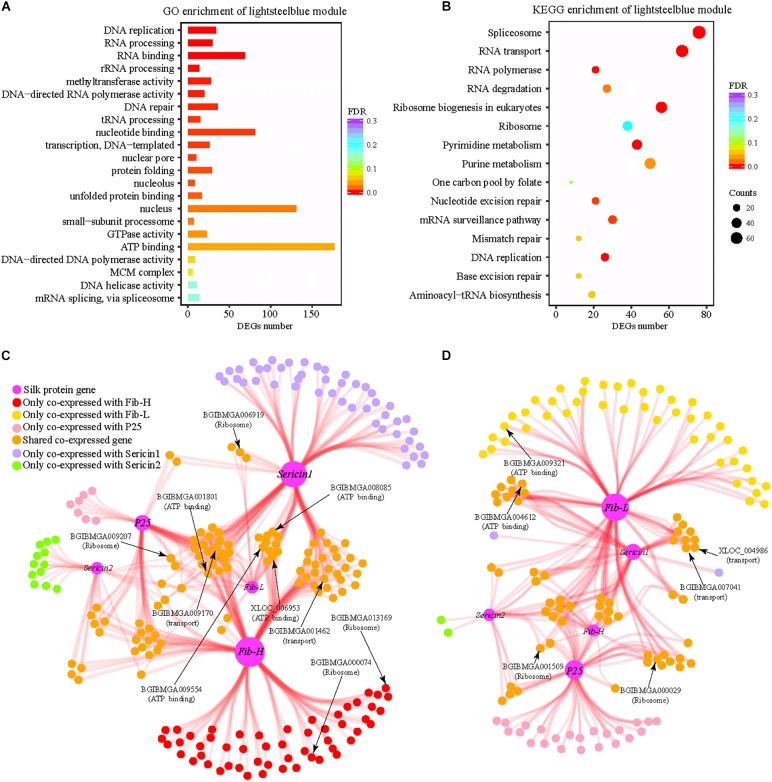
Functional enrichment of “lightsteelblue” module and differential co-expression network of silk-coding genes. **(A)** Gene ontology enrichment of “lightsteelblue” module. **(B)** KEGG enrichment of “lightsteelblue” module. **(C)** The co-expression network of silk-coding genes in the domestic silkworm. **(D)** The co-expression network of silk-coding genes in wild silkworm.

We have identified 3,453 DEGs which are differential expression at least at one stage and found that fibroin genes present higher expression levels in the domestic silkworm than the wild silkworm. To identify the DEGs that are closely co-expressed with silk-coding genes, we performed co-expression analysis. A total of 32 consistent modules are detected in the domestic and wild silkworms. We found that the genes in the “lightsteelblue” module (a sub-network) are enriched in functions including protein folding, biological energy, ribosome, and RNA transport (Figures 4A,B). These functions show concordance with the functional divergence in the silk gland between the domestic and wild silkworms (Figure 2). Interestingly, fibroin genes (*Fib-H*, *Fib-L*, and *P25*) and sericin genes (*Sericin1* and *Sericin2*) were also in the “lightsteelblue” module. In the “lightsteelblue” module of the domestic silkworm, we detected 400 DEGs co-expressed with silk-encoding genes, such as ribosome (BGIBMGA008335 and BGIBMGA006919), RNA transport (BGIBMGA005438 and BGIBMGA001699) and oxidative phosphorylation (BGIBMGA007211) related genes, which were up-regulated in the domestic silkworm (Supplementary Table S5). For the wild silkworm, we detected 258 DEGs co-expressed with silk-coding genes. Some of them (BGIBMGA013791 and BGIBMGA000029) are related to ribosome and RNA transport and up-regulated in wild silkworms. After removing the genes that show no differences in co-expression with silk-coding genes between the domestic and wild silkworms, the specific co-expression networks of silk-coding genes and DEGs are constructed for the domestic and wild silkworms, respectively (Figures 4C,D).

For the domestic silkworm, 142 DEGs are co-expressed with fibroin gene *Fib-H*. Among them, 53 genes are solely co-expressed with *Fib-H* (Figure 4C). One hundred and nineteen DEGs are co-expressed with *Sericin1* in the domestic silkworm and most of them are commonly co-expressed with *Fib-H* (Figure 4C). *Fib-L*, *P25*, and *Sericin2* are co-expressed with 29, 50, and 28 DEGs, respectively. Most DEGs co-expressed with *Fib-L*, *P25*, and *Sericin1* are shared by *Fib-H* and *Sericin1*. Moreover, the DEGs in the network are associated with functions including ATP-binding (e.g., BGIBMGA009554 and BGIBMGA008085), transport (e.g., BGIBMGA009170 and BGIBMGA001462), and ribosome (e.g., BGIBMGA006919 and BGIBMGA000074) (Figure 4C and Supplementary Table S6). Especially, two protein-coding genes (BGIBMGA000074 and BGIBMGA013169) related to the ribosome are co-expressed with *Fib-H*. Strikingly, the topology of the network of wild silkworm is quite different from that of the domestic silkworm (Figures 4C vs. D) and the network of the wild silkworm is much smaller than that of the domestic silkworm. Only 19 DEGs are co-expressed with *Fib-H* and no ribosome gene is linked to the *Fib-H* in the wild silkworm (Figure 4D). Less co-expressed genes are shown for the Wild silkworm *Fib-L* and *P25* in comparison to the domestic silkworm (Figure 4D). In addition, some co-expressed genes shared by silk-coding genes are involved in the ATP-binding, transport, and ribosome in the wild silkworm, but the number is less than that in the domestic silkworm (Figures 4C,D). These results demonstrate that divergence in biological energy, transport, and ribosome pathways might result in the difference in silk yield between the domestic and wild silkworms.

**FIGURE 5:**
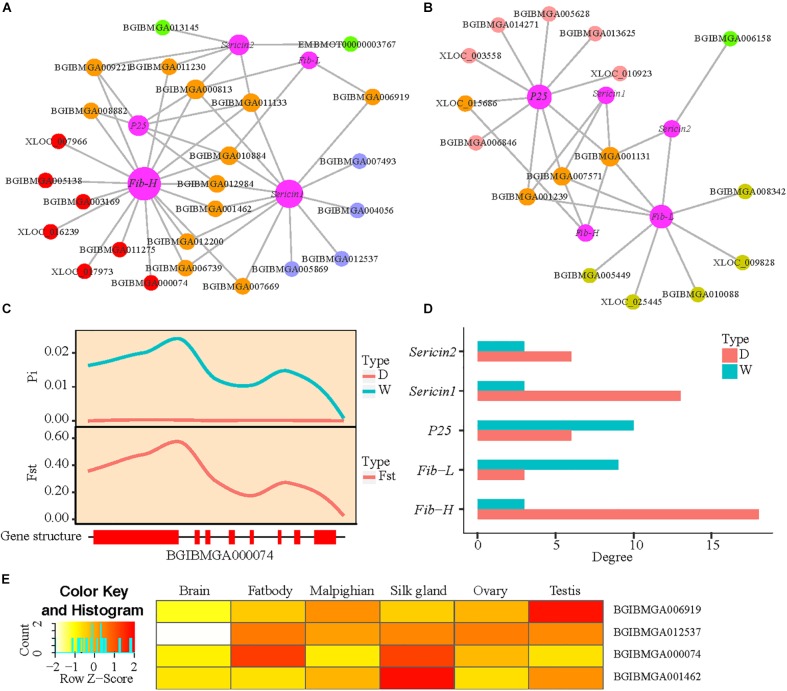
The influence of domestication on the co-expression network of silk-coding genes. **(A,B)** Domestic and differential co-expression network of silk-coding genes in the domestic and wild silkworms, respectively. **(C)** Nucleotide polymorphism (π) and Fixation index (*Fst*) of BGIBMGA000074. Red rectangle, exon. **(D)** Degree of five silk-coding genes in the domestic and differential co-expression network of the domestic and wild silkworm. **(E)** Tissue expression patterns of BGIBMGA006919, BGIBMGA012537, BGIBMGA000074, and BGIBMGA001462.

To further test the influence of domestication, the whole-genome sequencing data of the domestic and wild silkworm populations are used to identify genomic regions under artificial selection. After removing the genes without selection signature from the co-expression network, two small co-expression networks of silk-coding genes are constructed in the domestic and wild silkworms, respectively (Figures 5A,B). Interestingly, three genes with selection signatures (BGIBMGA000074, BGIBMGA012537, and BGIBMGA006919) related to ribosome biogenesis are found in the network of the domestic silkworm (Figure 5A). BGIBMGA000074 is especially co-expressed with *Fib-H*. As an example, BGIBMGA000074 exhibits a lower nucleotide polymorphism in the domestic silkworm than that in wild silkworm, and large population differentiation (*Fst* > 0.3497) between the domestic and wild silkworms (Figure 5C). BGIBMGA006919 is shared by *Fib-L* and *Sericin1*. BGIBMGA012537 is specifically co-expressed with the *Sericin1*. However, the ribosome genes were not detected in the small network of wild silkworms (Figure 5B). These results suggest that ribosome genes may be subjected to artificial selection during silkworm domestication. Apart from the ribosome genes, we also found that BGIBMGA001462, which relates to transport function, is co-expressed with *Fib-H* in the domestic silkworm (Figure 5A). Furthermore, this gene exhibits a specifical expression in the silk gland (Figure 5E). *Fib-H* is the largest protein molecule in the fibroin and the most important gene for silk production (Zhou et al., 2000). Strikingly, we found that *Fib-H* in the network of the domestic silkworm has more links than that of wild silkworm and *Sericin1* also presents a similar pattern (Figures 5A,B,D). These indicate that artificial selection during silkworm domestication might have directly acted not only on the silk-coding genes but also on silk production-related genes such as genes involved in biological energy, transport, and ribosome pathway.

## Discussion

After long-term artificial selection of domestication, the larval weight of the domestic silkworm is about a quadruple increase in comparison to the wild silkworm, no matter in the male or female (Supplementary Figure S1A). However, silk yield (CSW) of the domestic silkworm is nine times more than that of the wild silkworm (Supplementary Figure S1D). The correlation analysis indicates that the silk yield is largely dependent on the larval weight in the wild silkworm (Supplementary Figures S9A,B). Nevertheless, after adjusting the silk production with the larval weight, the domestic silkworm still has about twofold silk yields in comparison to the wild silkworm (Supplementary Figure S1E). This suggests that the high efficiency of silk protein synthesis and/or other mechanisms led to an increase in the silk yield of the domestic silkworm.

Indeed, our analysis for time-series comparative transcriptomes between the domestic and wild silkworm revealed some dynamic patterns of functional divergence during silk gland development. At early and intermediate stages of silk gland development, the up-regulated genes of the domestic silkworm mainly referred to DNA integration, nucleic acid binding, and transporter activity (Figure 2A), which are involved in cell growth and division (Oh and Irvine, 2008, 2009; Zhang et al., 2012). In fact, the silk gland of the domestic silkworm is larger than that of the wild silkworm (Figure 1). As the “factory” for fibroins synthesis, the cell number of the PSG is significantly increased in the domestic silkworm (Supplementary Figure S7C). That is, the domestic silkworm has many more “factories” than the wild silkworm to synthesize more silk fibroins. Apart from more “factories” for silk fibroins production, our results revealed that in the late stage of silk gland development, the up-regulated genes in the domestic silkworm are enriched in protein processing in the endoplasmic reticulum and ribosome pathways (Figure 2A and Supplementary Figure S4A), indicating that protein synthesis is more active in the domestic silkworm. These results are consistent with previous studies (Fang et al., 2015; Li J. Y. et al., 2017; Zhou et al., 2018).

In this study, divergences in the expression pattern of silk-coding genes and the composition of silk proteins were investigated between domestic and wild silkworms. The results suggested that the synthetic capacity of fibroin proteins is increased but the synthetic capacity of sericin proteins is decreased during silkworm domestication (Figure 3 and Supplementary Figure S8). This may result from the different selection pressures acted upon different components of cocoon proteins. High yields in silk fibroin and low yields in sericin are always a target of long-term, strongly artificial and breeding selection in the domestic silkworm because of its economic value (Xia et al., 2014). In the domestic silkworm, the strongly artificial and breeding selection might contribute to increase the synthetic capacity of fibroin proteins and decrease the synthetic capacity of sericin proteins. However, for wild silkworm, the genes encoding both sericin and fibroin undergo natural selection as they function importantly in protecting the cocoon in wild conditions (Dai et al., 2019).

To systematically understand the molecular mechanisms of silk yield increase during domestication, we constructed co-expression networks of silk-coding genes in the domestic and wild silkworms, respectively. Strikingly, we found that the network co-expressed with silk-coding genes of the domestic silkworm is much larger than that of the wild silkworm (Figure 4). Furthermore, genes co-expressed with silk-coding genes in the domestic silkworm have been subjected to artificial selection and the number of them is larger than those in wild silkworm (Figures 5A,B). This implies that many rather than a few genes contribute to silk yield increase during silkworm domestication and improvement. A recent resequencing study suggested that the nitrogen metabolism pathway is the most significantly enriched in the domestication-associated gene sets (Xiang et al., 2018). In the involved pathway, three genes participate in the alanine, aspartate, and glutamate synthesis, and then provide necessary substances for the silk protein synthesis (Xiang et al., 2018). Our results do not include the same genes as the previous study. This may be due to the fact that our results are based on dynamic patterns of transcriptomes in the silk gland while the previous study was based on the static selection signature in the genomes. Importantly, our results show that artificial selection during silkworm domestication might directly act not only on the silk-coding genes but also on the silk production-related genes as those ones that are implicated in biological energy, transport, and the ribosome pathway. For example, one gene is involved in the transport pathway (BGIBMGA001462) and another in the ribosome pathway (BGIBMGA000074). Both of them are co-expressed with *Fib-H* in the co-expression networks of silk-coding genes in the domestic silkworm (Figure 5A) and relatively high gene expression levels are detected in the silk gland across the examined tissues (Figure 5E), which further indicated that the silk production-related genes are involved in the silk yield increase after silkworm domestication.

Many economically important traits of domestic animals, such as milk yield of dairy cows, egg production of chickens, as well as silk yield of the silkworm, exhibit continuous distribution in hybrid populations and are known as quantitative traits. These traits are generally thought to be controlled by multiple genes or loci. Therefore, in the beginning, an “infinitesimal” model was proposed to formalize the polygene background, which is also known as the polygenic model. This model states that quantitative traits are controlled by a very large number of genes and each gene has very small and equal allelic effects (Mackay, 2001). Later, the distribution of allelic effects was found to be more nearly an exponential distribution, as a few loci exert large effects while a large number of loci exhibit very smaller effects (Lynch and Walsh, 1998). With this model, it is difficult to recognize the effect of each gene under the general framework of classical quantitative genetics (Hu et al., 2012). Most importantly, the effects of genes affecting quantitative traits vary with different genetic and sexual backgrounds, and external environments. The heritability of quantitative traits and the proportion of the phenotypic variance which is caused by genetic factors typically ranges from ∼5 to 50% (Georges et al., 2019), even though evolution and the improvement of complex traits are involved in many genetic factors, including genotype by genotype interaction (epistasis), genotype by sex interaction, genotype by the environment, and pleiotropy. Thus, quantitative traits are also known as complex traits. Our results suggested that increase in silk yield during silkworm domestication has been involved in the improvement of a biological system which includes not only expansion of “factories” of protein synthesis but also high-expression of silk-coding genes as well as silk production-related genes such as biological energy, transport, and ribosome pathway genes. This is due to the property of genetic architecture of complex traits.

## Conclusion

In summary, we used a combination of comparative multi-omics and dynamic network biological methods to understand the genetic basis and possible molecular mechanisms of silk yield improvement in the domestic silkworm. In this sense, our study provides a methodology reference for investigating the molecular mechanisms of a complex trait formation in other domestic animals. In addition, our results provide a valuable resource for further understanding molecular insights into silk yield increase after silkworm domestication and breeding improvement.

## Data Availability Statement

The raw RNA-seq data is available at https://bigd.big.ac.cn/ under accession ID: PRJCA001835. 15 genome resequencing data are available in https://bigd.big.ac.cn/ under accession ID: PRJCA002125 and another three samples are available in https://www.ncbi.nlm.nih.gov/sra under the accession ID: SAMEA2357881 (Xiafang strain), SAMEA2357880 (Nanchong Sichuan), and SAMEA2357878 (Ankang Shanxi).

## Ethics Statement

Experiments were conducted in accordance with the protocol approved by the Institutional Animal Care and Use Committee of the Chongqing University (permit number CBE-A201607020).

## Author Contributions

ZZ and Q-YY participated in the research design. C-ZQ, H-BZ, S-SL, and C-JZ reared and collected the experiment materials. Q-ZZ, FP, S-SL, and C-JZ analyzed the data and performed the experiments. Q-ZZ and PF wrote the manuscript. ZZ and Q-YY supervised and revised the manuscript. All authors read and approved the final manuscript.

## Conflict of Interest

The authors declare that the research was conducted in the absence of any commercial or financial relationships that could be construed as a potential conflict of interest.

## Supplementary Material

FIGURE S1 Phenotype variation between the domestic and wild silkworms. D, the domestic silkworm; W, the wild silkworm. **(A)** Larval weight (LW) in the early wandering stage. **(B)** Whole cocoon weight (WCW). **(C)** Pupae weight (PW). **(D)** Cocoon shell weight (CSW). **(E)** The ratio of CSW to WL. **(F)** The ratio of CSW to WCW (%). Asterisk represent the Wilcoxon-test *P-*value < 0.05.

FIGURE S2 Box plot of expression profile of all genes in the developmental process of silk gland between the domestic and wild silkworms.

FIGURE S3 Expression levels of transport and ECM genes that are up-regulated in the domestic silkworm at least in five time points. **(A)** Up-regulated genes in relation to transporter activity. **(B)** Up-regulated genes in the ECM pathway.

FIGURE S4 KEGG enrichment of the differentially expressed genes between the domestic and wild silkworms. **(A)** KEGG enrichment of up-regulated genes in the domestic silkworm. **(B)** KEGG enrichment of down-regulated genes in the domestic silkworm.

FIGURE S5 The GO and KEGG terms exhibit higher enrichment in the down-regulated genes than up-regulated genes at all examined time points.

FIGURE S6 Expression of ribosome genes up-regulated in the domestic silkworm at least in five time points.

FIGURE S7 Immune staining and histological observations of the posterior silk gland. **(A,B)** Immune staining the posterior silk gland in the domestic and wild silkworm. **(C)** Cell number of posterior silk gland in the domestic and wild silkworm. (Asterisk represent *t*-test *P*-value < 0.05).

FIGURE S8 The gene expression correlation of fibroin genes between RNA-seq and qRT-PCR. Gene expression correlation between RNA-seq and qRT-PCR for fibroin genes, *Fib-H* **(A)**, *Fib-L* **(B)**, and *P25* **(C)**.

FIGURE S9 Correlation between larval weight and cocoon shell weight. **(A)** Larval weight correlated with cocoon shell weight in the domestic silkworm. **(B)** Larval weight correlated with cocoon shell weight in the wild silkworm.

TABLE S1 The primer sequences for qRT-PCR validation experiment.

TABLE S2 Summary of clean RNA-seq data mapped to the silkworm reference genome.

TABLE S3 Expression levels of all the genes across 7 developmental stages of silk gland in the domestic and wild silkworms. Gene expression was normalized by FPKM.

TABLE S4 Distribution of gene expression in the developmental process of silk gland between the domestic and wild silkworms.

TABLE S5 Differentially expressed genes between the domestic and wild silkworms.

TABLE S6 Annotation of DEGs in the specific co-expression network of silk-coding genes in the domestic and wild silkworm, separately.

## Abbreviations

CSW: cocoon shell weight
DAPI: 4′,6-diamidino-2-phenylindole
DEG: differentially expressed gene
ECM: extracellular matrix
*Fib-H*: fibroin heavy chain
*Fib-L*: fibroin light chain
GO: gene ontology
KEGG: Kyoto Encyclopedia of Genes and Genomes
MSG: middle silk gland
PSG: posterior silk gland
XF: Xiafang strain.
