# Supplementary figures and images for: A Comparison of Co-expression Networks in Silk Gland Reveals the Causes of Silk Yield Increase During Silkworm Domestication
Source: Front Genet. 2020 Mar 27;11:225. doi: 10.3389/fgene.2020.00225 (PMC7119365; doi:10.3389/fgene.2020.00225)

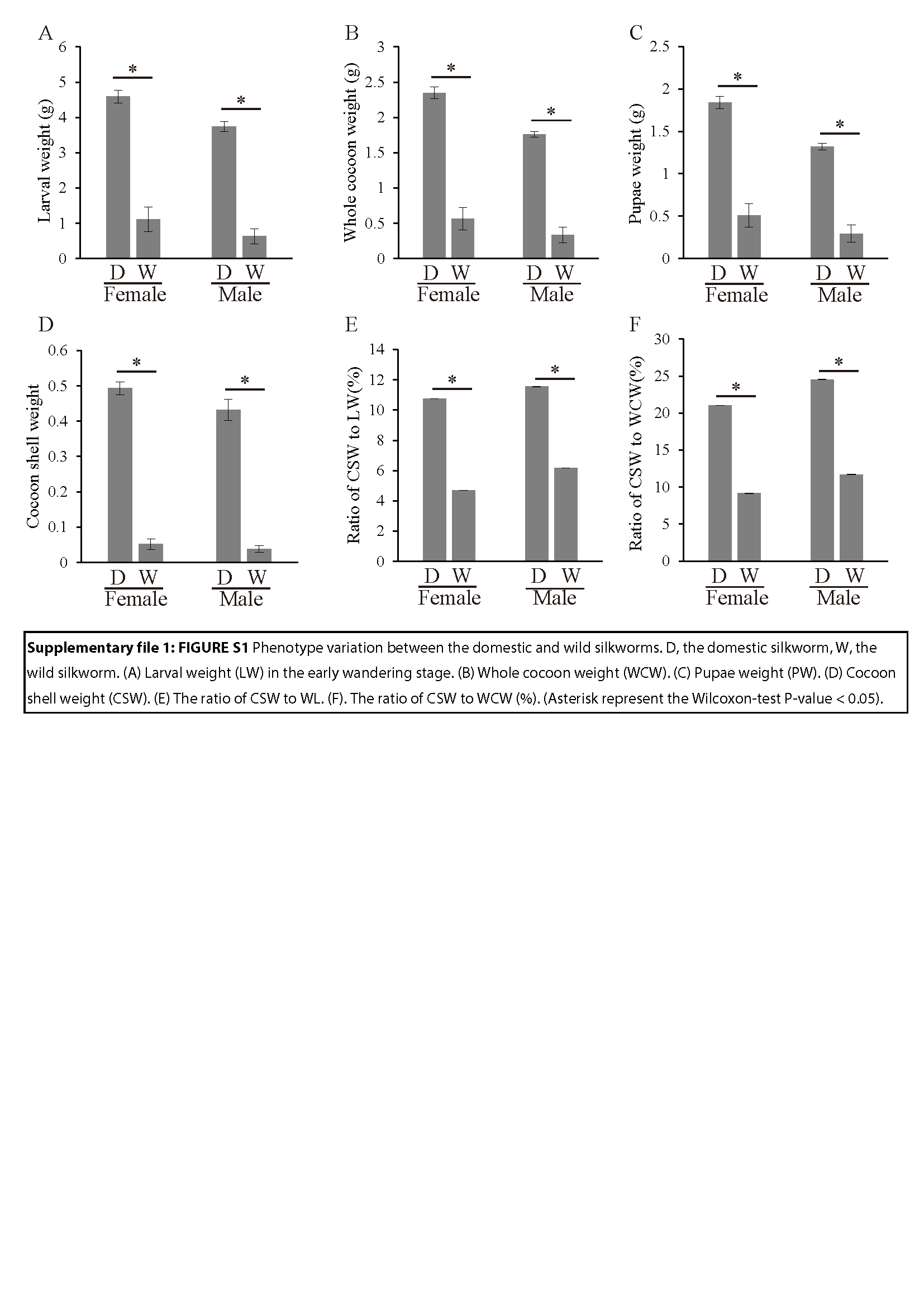

Supplement: FIGURE S1 — Phenotype variation between the domestic and wild silkworms. D, the domestic silkworm; W, the wild silkworm. (A) Larval weight (LW) in the early wandering stage. (B) Whole cocoon weight (WCW). (C) Pupae weight (PW). (D) Cocoon shell weight (CSW). (E) The ratio of CSW to WL. (F) The ratio of CSW to WCW (%). Asterisk represent the Wilcoxon-test P-value < 0.05. [file Image_1.tif]

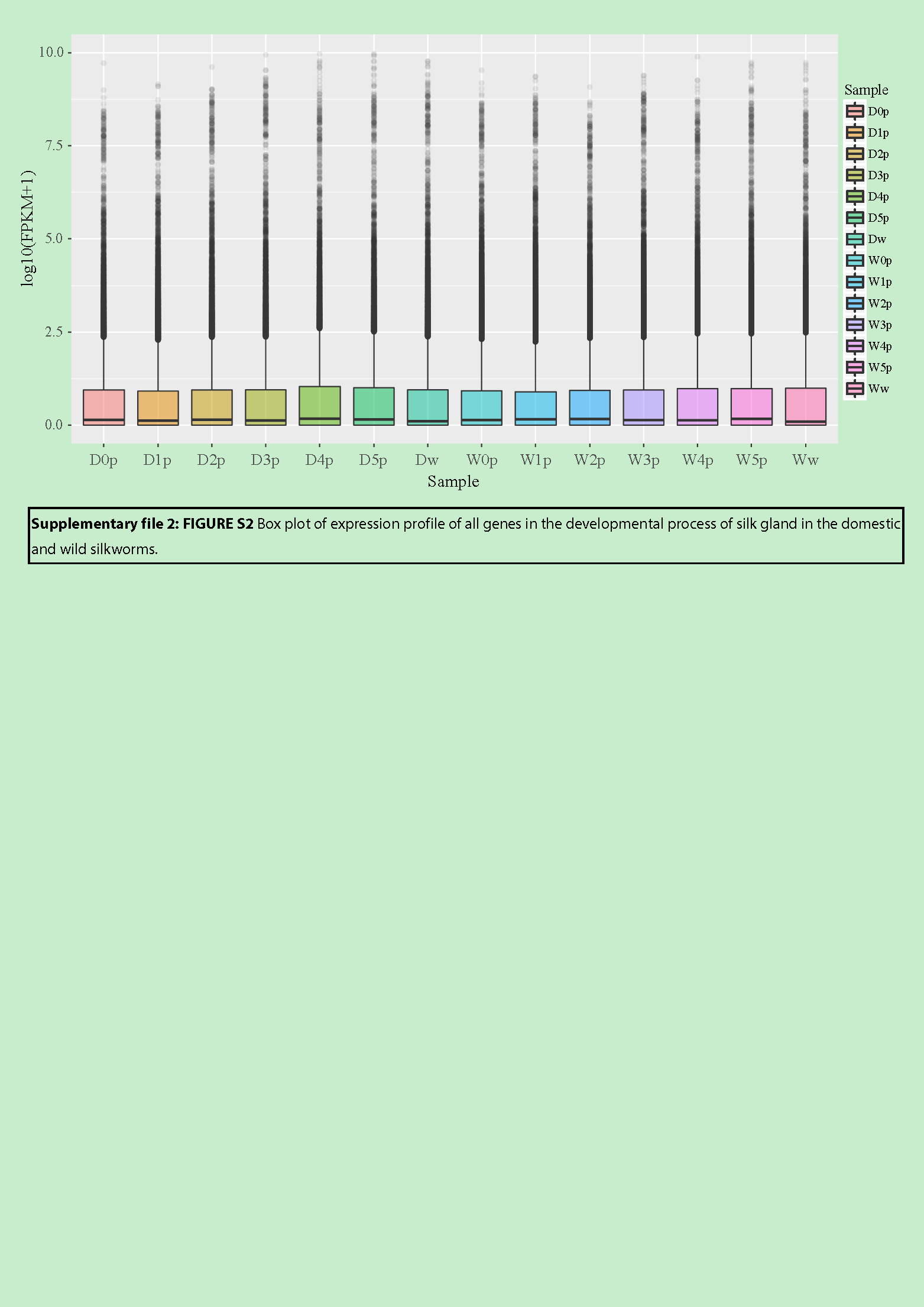

Supplement: FIGURE S2 — Box plot of expression profile of all genes in the developmental process of silk gland between the domestic and wild silkworms. [file Image_2.TIF]

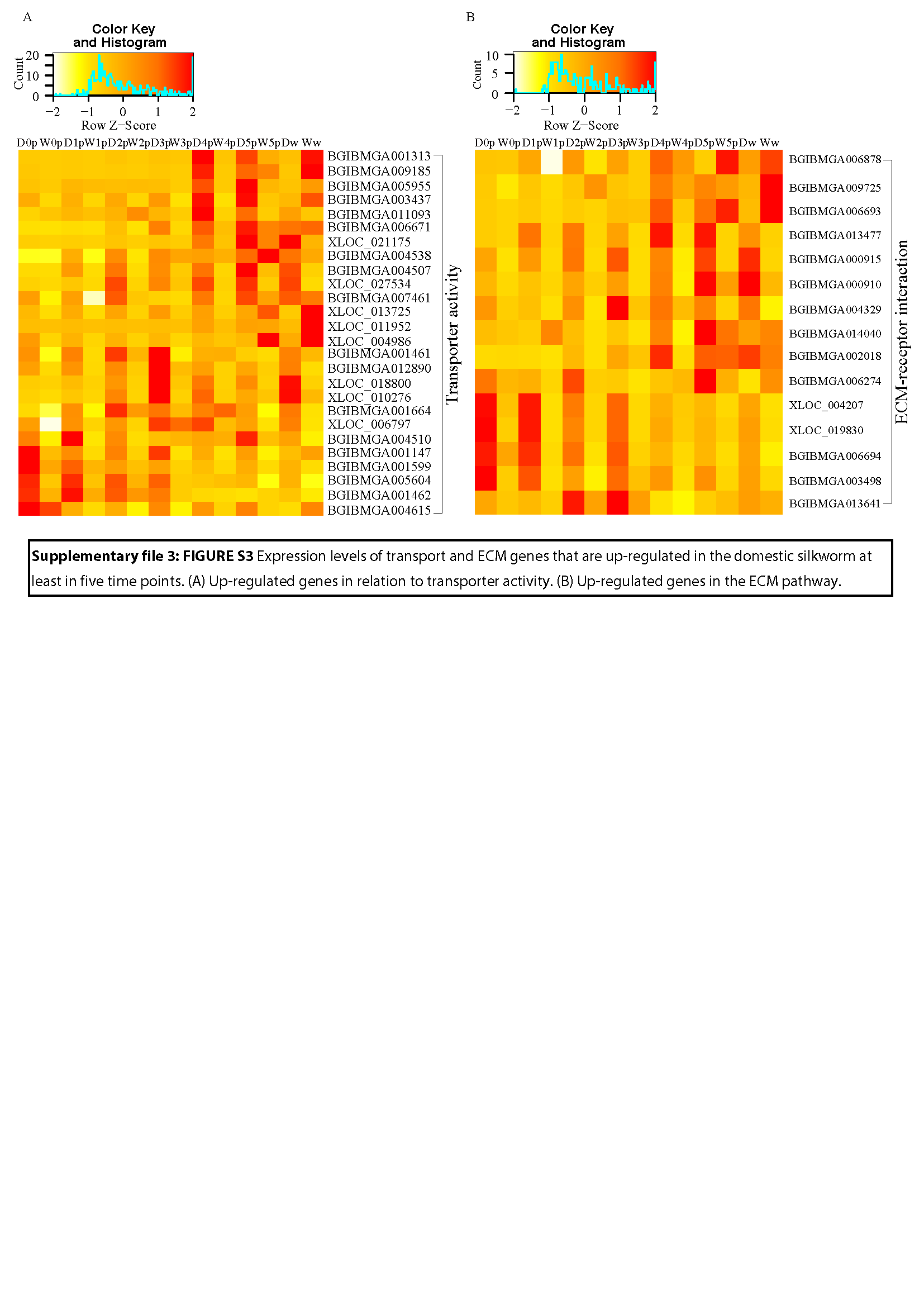

Supplement: FIGURE S3 — Expression levels of transport and ECM genes that are up-regulated in the domestic silkworm at least in five time points. (A) Up-regulated genes in relation to transporter activity. (B) Up-regulated genes in the ECM pathway. [file Image_3.TIF]

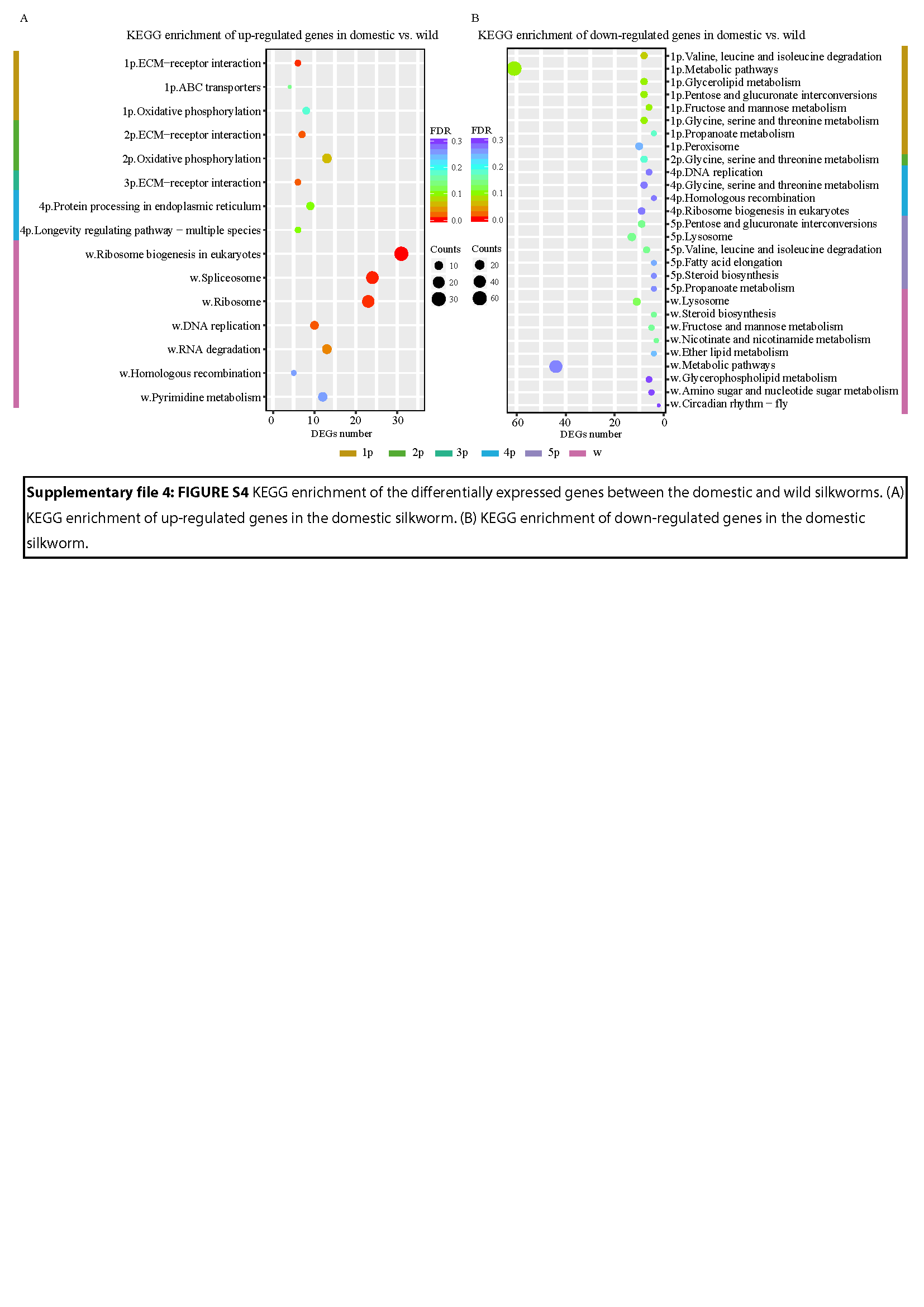

Supplement: FIGURE S4 — KEGG enrichment of the differentially expressed genes between the domestic and wild silkworms. (A) KEGG enrichment of up-regulated genes in the domestic silkworm. (B) KEGG enrichment of down-regulated genes in the domestic silkworm. [file Image_4.tif]

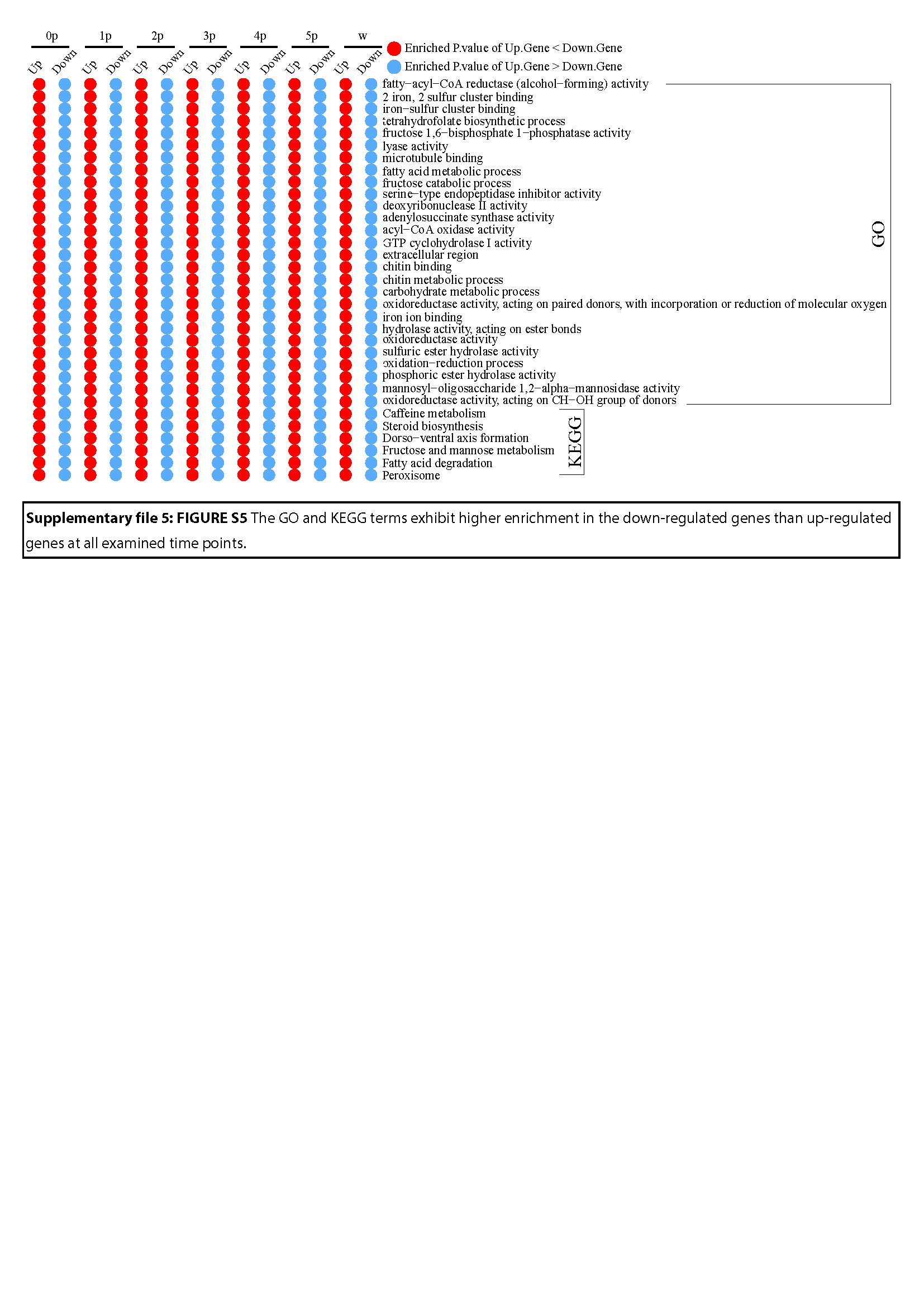

Supplement: FIGURE S5 — The GO and KEGG terms exhibit higher enrichment in the down-regulated genes than up-regulated genes at all examined time points. [file Image_5.TIF]

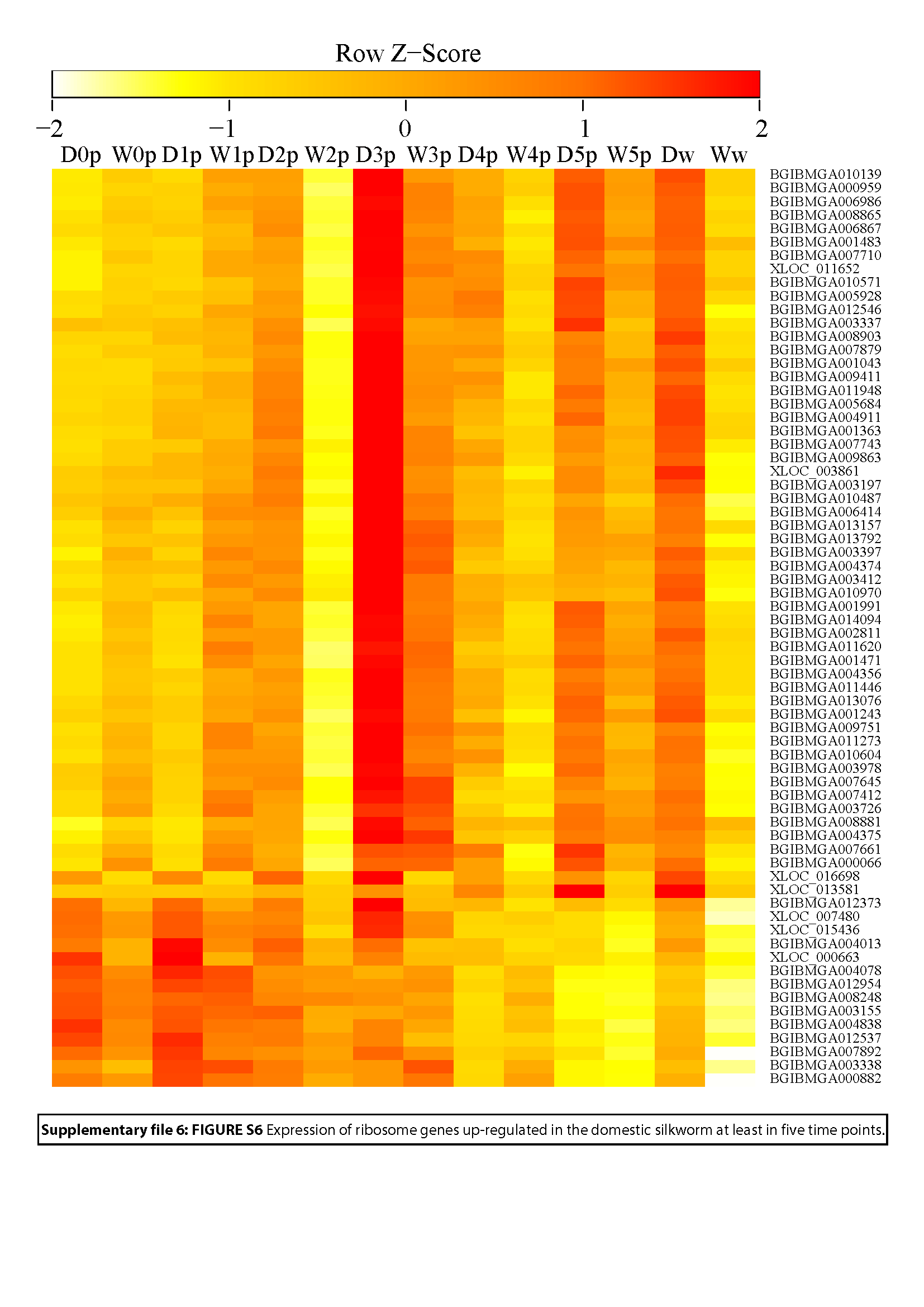

Supplement: FIGURE S6 — Expression of ribosome genes up-regulated in the domestic silkworm at least in five time points. [file Image_6.TIF]

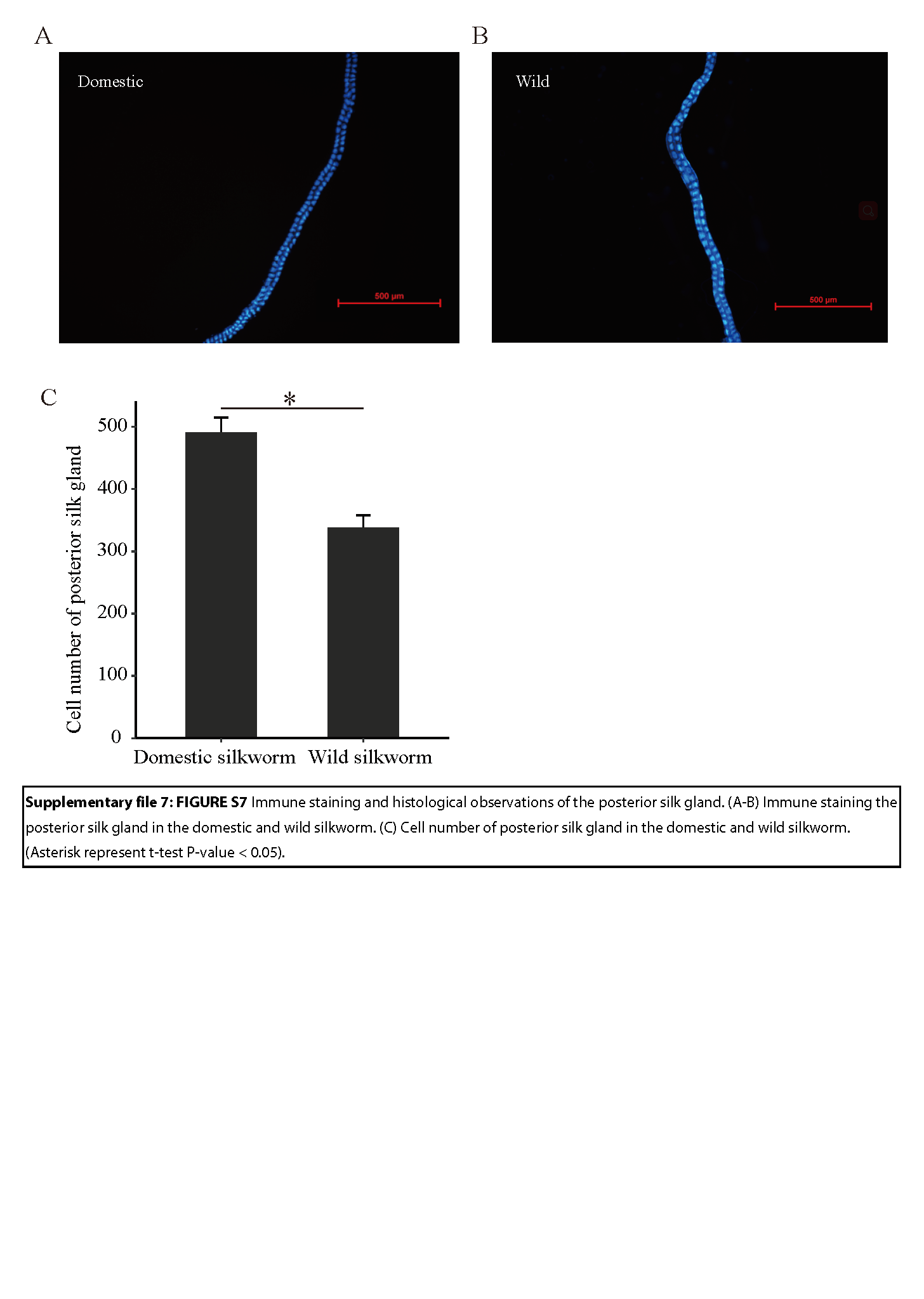

Supplement: FIGURE S7 — Immune staining and histological observations of the posterior silk gland. (A,B) Immune staining the posterior silk gland in the domestic and wild silkworm. (C) Cell number of posterior silk gland in the domestic and wild silkworm. (Asterisk represent t-test P-value < 0.05). [file Image_7.tif]

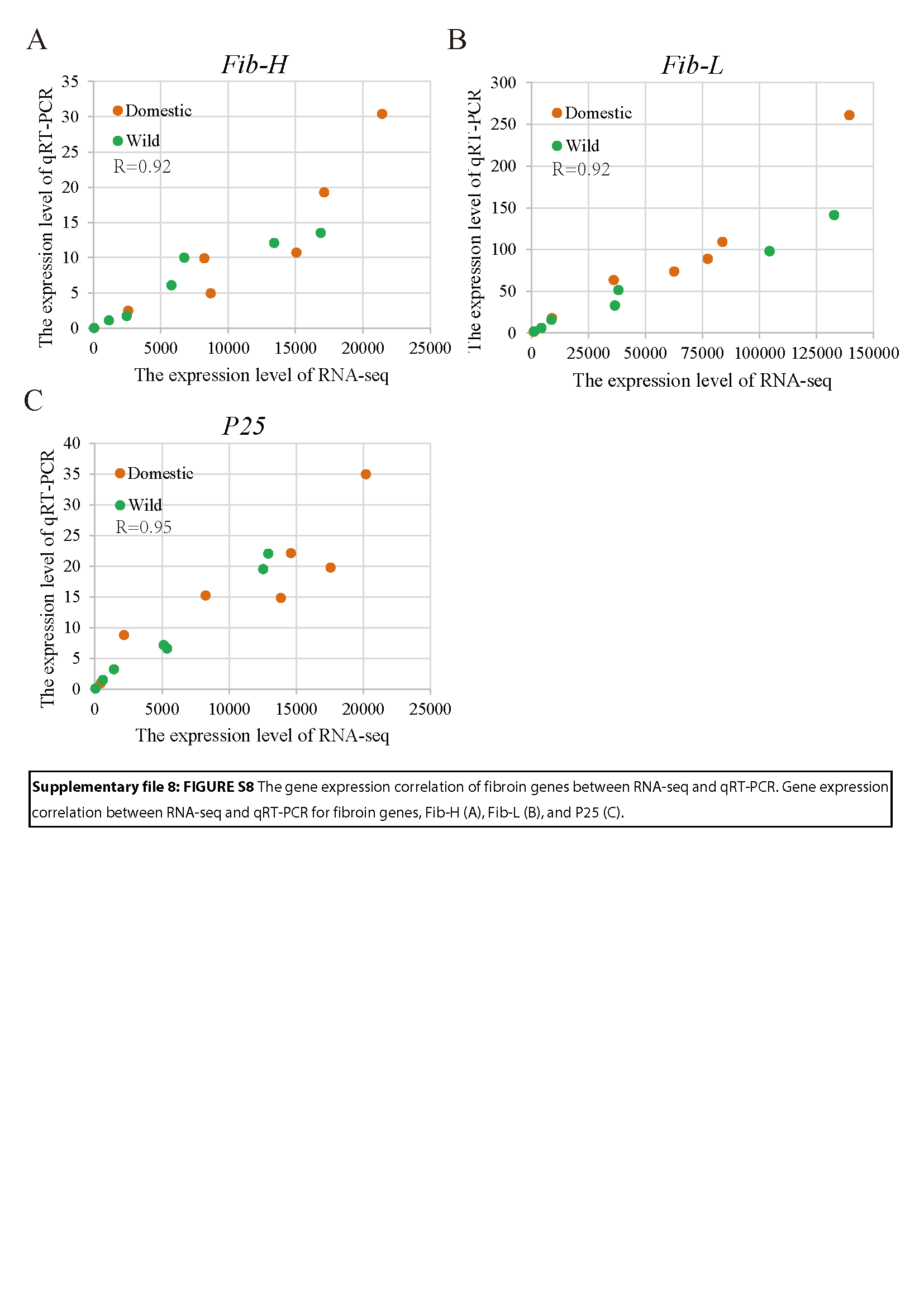

Supplement: FIGURE S8 — The gene expression correlation of fibroin genes between RNA-seq and qRT-PCR. Gene expression correlation between RNA-seq and qRT-PCR for fibroin genes, Fib-H (A), Fib-L (B), and P25 (C). [file Image_8.tif]

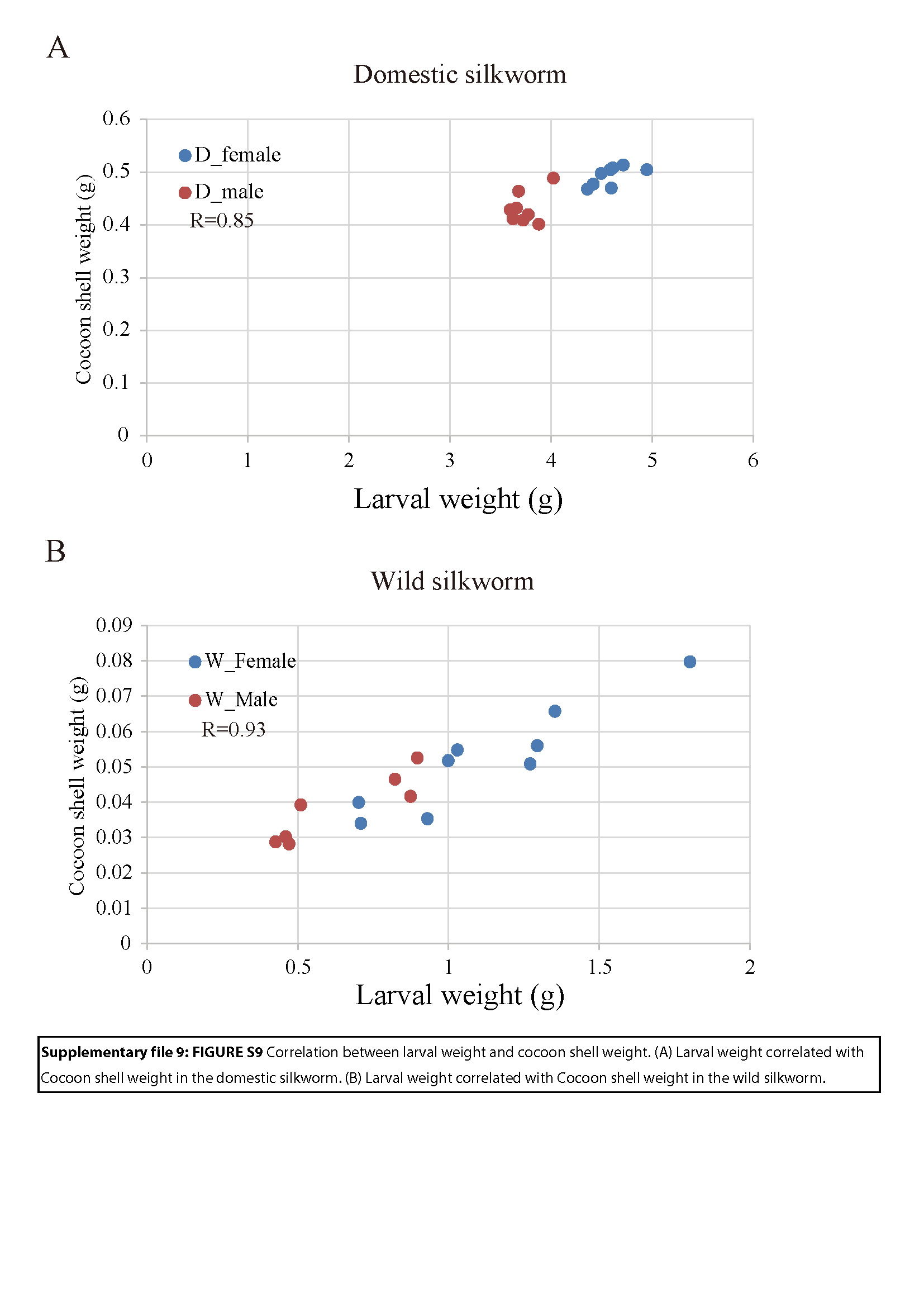

Supplement: FIGURE S9 — Correlation between larval weight and cocoon shell weight. (A) Larval weight correlated with cocoon shell weight in the domestic silkworm. (B) Larval weight correlated with cocoon shell weight in the wild silkworm. [file Image_9.tif]
