# Supplementary material for: A Comparison of Co-expression Networks in Silk Gland Reveals the Causes of Silk Yield Increase During Silkworm Domestication
Source: Front Genet. 2020 Mar 27;11:225. doi: 10.3389/fgene.2020.00225 (PMC7119365; doi:10.3389/fgene.2020.00225)
Supplement: TABLE S1 — The primer sequences for qRT-PCR validation experiment. [file Table_1.DOC]

Table S1 The primer sequences for qRT-PCR validation experiment

| **Gene** | **Left primer sequences** | **Right primer sequences** |
| --- | --- | --- |
| *Fib-H* | TTGTGATCTTGTGCTGCGCT | TCGATAACTGCCCCAGATGC |
| *Fib-L* | CGATACTCTGTCGGACCAGC | TGAGCGGTTATGTAGGCAGC |
| *P25* | GGGTCTGCCCATCTTCCA | TCGCCAGCCAGTTCCTCT |
| *RPL3* | CGGTGTTGTTGGATACATTGAG | GCTCATCCTGCCATTTCTTACT |
