# Supplementary material for: A Comparison of Co-expression Networks in Silk Gland Reveals the Causes of Silk Yield Increase During Silkworm Domestication
Source: Front Genet. 2020 Mar 27;11:225. doi: 10.3389/fgene.2020.00225 (PMC7119365; doi:10.3389/fgene.2020.00225)
Supplement: TABLE S2 — Summary of clean RNA-seq data mapped to the silkworm reference genome. [file Table_2.doc]

Table S2 Summary of clean RNA-seq data mapped to the silkworm reference genome

| **Sample** | **Total clean reads** | **Left reads** | **Right reads** | **Uniquely mapped reads** | **Multiple mapped reads** | **Total mapped reads** |
| --- | --- | --- | --- | --- | --- | --- |
| D0p1 | 40 147 950 | 16 671 070 (83.05%) | 15 840 573 (78.91%) | 31 305 574 (77.98%) | 1 206 069 (3.00%) | 32 511 643 (80.98%) |
| D0p2 | 43 794 908 | 18 135 325 (82.82%) | 17 085 680 (78.03%) | 33 735 838 (77.03%) | 1 485 167 (3.39%) | 35 221 005 (80.42%) |
| D1p1 | 45 433 030 | 18 147 902 (79.89%) | 16 603 797 (73.09%) | 32 249 382 (70.98%) | 2 502 317 (5.51%) | 34 751 699 (76.49%) |
| D1p2 | 40 605 238 | 16 556 371 (81.55%) | 15 023 532 (74.00%) | 29 524 216 (72.71%) | 2 055 687 (5.06%) | 31 579 903 (77.77%) |
| D2p1 | 41 622 064 | 15 880 812 (76.31%) | 13 873 632 (66.66%) | 26 633 829 (63.99%) | 3 120 615 (7.50%) | 29 754 444 (71.49%) |
| D2p2 | 36 536 002 | 14 147 569 (77.44%) | 12 370 651 (67.72%) | 24 168 978 (66.15%) | 2 349 242 (6.43%) | 26 518 220 (72.58%) |
| D3p1 | 38 541 980 | 14 915 798 (77.40%) | 13 161 490 (68.30%) | 25 274 293 (65.58%) | 2 802 995 (7.27%) | 28 077 288 (72.85%) |
| D3p2 | 39 635 704 | 15 419 962 (77.81%) | 13 577 108 (68.51%) | 26 245 738 (66.22%) | 2 751 332 (6.94%) | 28 997 070 (73.16%) |
| D4p1 | 42 974 866 | 15 860 267 (73.81%) | 13 516 931 (62.91%) | 25 552 809 (59.46%) | 3 824 389 (8.90%) | 29 377 198 (68.36%) |
| D4p2 | 38 724 276 | 14 776 366 (76.32%) | 12 839 149 (66.31%) | 24 129 823 (62.31%) | 3 485 692 (9.00%) | 27 615 515 (71.31%) |
| D5p1 | 46 492 302 | 16 850 092 (72.49%) | 14 393 416 (61.92%) | 27 134 752 (58.36%) | 4 108 756 (8.84%) | 31 243 508 (67.20%) |
| D5p2 | 45 907 158 | 16 923 179 (73.73%) | 14 238 127 (62.03%) | 26 834 891 (58.45%) | 4 326 415 (9.42%) | 31 161 306 (67.88%) |
| Dw | 44 977 760 | 16 613 120 (73.87%) | 14 142 726 (62.89%) | 26 965 662 (59.95%) | 3 790 184 (8.43%) | 30 755 846 (68.38%) |
| W0p1 | 47 166 250 | 17 658 923 (74.88%) | 16 447 153 (69.74%) | 33 004 788 (69.98%) | 1 101 288 (2.33%) | 34 106 076 (72.31%) |
| W0p2 | 44 102 966 | 16 914 015 (76.70%) | 16 037 617 (72.73%) | 31 768 918 (72.03%) | 1 182 714 (2.68%) | 32 951 632 (74.72%) |
| W1p1 | 45 744 868 | 17 302 944 (75.65%) | 16 372 144 (71.58%) | 32 264 901 (70.53%) | 1 410 187 (3.08%) | 33 675 088 (73.62%) |
| W1p2 | 39 923 882 | 15 281 261 (76.55%) | 13 999 742 (70.13%) | 28 051 976 (70.26%) | 1 229 027 (3.08%) | 29 281 003 (73.34%) |
| W2p1 | 53 392 084 | 20 028 739 (75.03%) | 18 390 318 (68.89%) | 36 091 327 (67.60%) | 2 327 730 (4.36%) | 38 419 057 (71.96%) |
| W2p2 | 56 196 984 | 20 984 942 (74.68%) | 19 158 371 (68.18%) | 37 479 313 (66.69%) | 2 664 000 (4.74%) | 40 143 313 (71.43%) |
| W3p1 | 47 406 446 | 17 594 474 (74.23%) | 15 882 572 (67.01%) | 31 642 289 (66.75%) | 1 834 757 (3.87%) | 33 477 046 (70.62%) |
| W3p2 | 40 792 232 | 13 532 738 (66.35%) | 12 180 583 (59.72%) | 23 536 276 (57.70%) | 2 177 045 (5.34%) | 25 713 321 (63.03%) |
| W4p | 46 866 636 | 16 618 465 (70.92%) | 14 705 579 (62.76%) | 28 168 102 (60.10%) | 3 155 942 (6.73%) | 31 324 044 (66.84%) |
| W5p1 | 38 941 510 | 9 842 241 (50.55%) | 8 513 129 (43.72%) | 16 003 513 (41.10%) | 2 351 857 (6.04%) | 18 355 370 (47.14%) |
| W5p2 | 45 515 564 | 13 117 479 (57.64%) | 11 161 107 (49.04%) | 21 293 342 (46.78%) | 2 985 244 (6.56%) | 24 278 586 (53.34%) |
| Ww | 53 793 662 | 18 651 075 (69.34%) | 15 656 295 (58.21%) | 29 550 181 (54.93%) | 4 757 189 (8.84%) | 34 307 370 (63.78%) |
