# Supplementary material for: A Comparison of Co-expression Networks in Silk Gland Reveals the Causes of Silk Yield Increase During Silkworm Domestication
Source: Front Genet. 2020 Mar 27;11:225. doi: 10.3389/fgene.2020.00225 (PMC7119365; doi:10.3389/fgene.2020.00225)
Supplement: TABLE S4 — Distribution of gene expression in the developmental process of silk gland between the domestic and wild silkworms. [file Table_4.doc]

Table S4 Distribution of gene expressions in the silk gland between domestic and wild silkworms

| **Interval**  **Sample** | 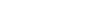 | 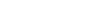 | 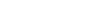 | 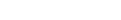 | 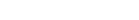 | 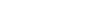 |
| --- | --- | --- | --- | --- | --- | --- |
| D0p | 20 693 (69.69%) | 5 092 (17.15%) | 2 548 (8.58%) | 1 028 (3.46%) | 235 (0.79%) | 95 (0.32%) |
| D1p | 20 941 (70.53%) | 4 758 (16.03%) | 2 485 (8.37%) | 1 126 (3.79%) | 281 (0.95%) | 100 (0.34%) |
| D2p | 20 730 (69.82%) | 4 956 (16.69%) | 2 579 (8.69%) | 1 028 (3.46%) | 280 (0.94%) | 118 (0.40%) |
| D3p | 20 713 (69.76%) | 4 885 (16.45%) | 2 627 (8.85%) | 1 061 (3.57%) | 282 (0.95%) | 123 (0.41%) |
| D4p | 20 086 (67.65%) | 5 349 (18.02%) | 2 851 (9.60%) | 1 018 (3.43%) | 266 (0.90%) | 121 (0.41%) |
| D5p | 20 260 (68.24%) | 5 355 (18.04%) | 2 718 (9.15%) | 970 (3.27%) | 268 (0.90%) | 120 (0.40%) |
| DW | 20 616 (69.44%) | 5 087 (17.13%) | 2 563 (8.63%) | 1 027 (3.46%) | 273 (0.92%) | 125 (0.42%) |
| W0p | 20 907 (70.42%) | 4 637 (15.62%) | 2 502 (8.43%) | 1 223 (4.12%) | 313 (1.05%) | 109 (0.37%) |
| W1p | 21 038 (70.86%) | 4 611 (15.53%) | 2 342 (7.89%) | 1 216 (4.10%) | 376 (1.27%) | 108 (0.36%) |
| W2p | 20 758 (69.91%) | 4 890 (16.47%) | 2 608 (8.78%) | 1 068 (3.60%) | 266 (0.90%) | 101 (0.34%) |
| W3p | 20 796 (70.04%) | 4 813 (16.21%) | 2 471 (8.32%) | 1 147 (3.86%) | 337 (1.14%) | 127 (0.43%) |
| W4p | 20 554 (69.23%) | 5 080 (17.11%) | 2 664 (8.97%) | 1 002 (3.37%) | 273 (0.92%) | 118 (0.40%) |
| W5p | 20 422 (68.78%) | 5 275 (17.77%) | 2 629 (8.85%) | 945 (3.18%) | 293 (0.99%) | 127 (0.43%) |
| Ww | 20 480 (68.98%) | 5 178 (17.44%) | 2 661 (8.96%) | 992 (3.34%) | 261 (0.88%) | 119 (0.40%) |
